# In Vitro Shear Bond Strength of Orthodontic Brackets after Enamel Conditioning with Acid Etching and Hydroabrasion

**DOI:** 10.3390/dj8040108

**Published:** 2020-09-30

**Authors:** Michele Tepedino, Maciej Iancu Potrubacz, Lorenzo Arrizza, Manuela Russo, Francesco Cavarra, Massimo Cordaro, Claudio Chimenti

**Affiliations:** 1 Department of Biotechnological and Applied Clinical Sciences, University of L’Aquila, Viale S. Salvatore Edificio Delta 6, 67100 L’Aquila, Italy; maciekip74@gmail.com (M.I.P.); manuela.russo4@gmail.com (M.R.); claudio.chimenti@cc.univaq.it (C.C.); 2 Fondazione Policlinico Universitario A. Gemelli IRCCS, Roma-Università Cattolica del Sacro Cuore, Istituto di Clinica Odontoiatrica e Chirurgia Maxillo-facciale, Largo Agostino Gemelli, 00168 Rome, Italy; massimo.cordaro@unicatt.it; 3 Center for Microscopy, University of L’Aquila, Via Vetoio 2, 67100 L’Aquila, Italy; lorenzo.arrizza@ing.univaq.it; 4 Section of Odontostomatology, Department of Clinical and Specialist Assistance, Sant’Andrea Hospital, Corso Mario Abbate, 13100 Vercelli, Italy; cavarrafr@gmail.com

**Keywords:** hydroabrasion, shear bond strength, orthodontic, enamel

## Abstract

The purpose of this study was to evaluate the shear bond strength and adhesive remnant index ARI) of orthodontic brackets following enamel conditioning with acid etching, hydroabrasion, and with both procedures. Thirty extracted human premolars were divided into three groups and received either acid etching, hydroabrasion or both procedures. Orthodontic brackets were bonded with composite resin. Shear bond strength was tested with a tensile machine, then the teeth were observed under a stereomicroscope to evaluate ARI scores. The enamel morphology after each conditioning method was evaluated with scanning electron microscope imaging. A one-way ANOVA and a Kruskal−Wallis H test were used to compare the bond strength and the ARI scores among the three groups. Hydroabrasion alone produced shear bond strength values below clinical acceptability, while the combination of acid etching and hydroabrasion produced the highest values. The ARI scores in the hydroabrasion group were significantly different from the other groups. Hydroabrasion followed by acid etching was effective in increasing the shear bond strength of orthodontic brackets. Further in vivo studies are needed to confirm the cost and benefits of this technique.

## 1. Introduction

When Buonocore in 1955 first described the use of phosphoric acid etching, it became possible to achieve a strong bond between resin composite and enamel [1]. Some years later, Newman used this technique for the bonding of orthodontic brackets [2]. Since then, enamel etching has been the gold standard for orthodontic bracket bonding: the phosphoric acid modifies the enamel surface into a typical etching pattern, allowing the mechanical interlocking of the adhesive into the created roughness [3]. To be considered ideal, an orthodontic bonding should be strong enough to prevent accidental failure, while minimizing the damage to the enamel and being reversible, allowing the removal of the bracket without significant damage to the tooth [4]. Phosphoric acid etching provides a good bond strength, but on the other hand is considered by many authors as having an iatrogenic effect [5,6]. Some studies revealed that etching leads to a loss of 10–30 μm of enamel [7], and that sometimes cracks are observed, which can even reach a depth of 80 μm [3]. For this reason, alternative methods have also been studied, like the use of maleic and polyacrylic acid; however, this resulted in a reduction in bond strength [8,9]. Another method proposed is air-abrasion, a technology that uses a high-speed beam of solid particles propelled by air pressure to obtain a mechanical roughening of the enamel surface. A study revealed that the enamel loss obtained after air-abrasion is equal to or even less than that of chemical etching [3], but contradicting results have been reported regarding bond strength: van Waveren Hogervorst et al. [3] reported that air-abrasion alone is not an adequate technique for orthodontic bonding; other authors demonstrated that air-abrasion in addition to acid etching is able to increase the bond strength [10,11], while other studies contradicted those findings [12,13,14,15]. However, this large variation of results could be explained by differences in particle size, air pressure, distance and duration of the application [16]. Air-abrasion has some drawbacks, mostly due to the dispersion of aluminum oxide particles in the patient’s mouth and around the operative field, which requires the use of protective eyewear for both the operator and the patient: such limits have been overcome by hydroabrasion, a technique where the powder is propelled by an air and water spray, which helps control the dissemination of the aluminum particles [17]. Hydroabrasion has already been described as a viable method for composite removal after orthodontic debonding [18], but to the knowledge of the authors of the present work, no data are available regarding its use as a method for enamel conditioning before orthodontic bonding of metal brackets. Therefore, the aim of the present study was to evaluate in vitro the shear bond strength and the adhesive remnant index on extracted teeth after three methods of enamel conditioning: acid etching; hydroabrasion; and a combination of hydroabrasion and acid etching. The null hypothesis was that no difference exists between the three methods of enamel conditioning.

## 2. Materials and Methods

The present research protocol was approved by the Internal Review Board of the University of L’Aquila, Italy (Protocol no 25403, 22/2018, 6th June 2018): the need for the informed consent was waived by the Internal Review Board, and all methods were performed in accordance with the relevant guidelines and regulations.

Thirty-three upper premolars extracted for therapeutic reasons, such as periodontal problems or orthodontic treatment, were selected after being screened under 2.5X magnifying loupes for presenting an intact enamel surface and no signs of erosion or abrasion, no surface demineralization, no decay and no traumatic damage provoked by forceps during the extraction procedure. The teeth were disinfected with 5% sodium hypochlorite solution (Niclor 5, Ogna, Muggiò, Italy), then stored in 0.9% sodium chloride solution at room temperature until the start of the experimentation and for no more than 2 months.

The 33 selected teeth were coded with a number impressed on the root, then divided into three groups of ten premolars each, by generating a random sequence number with an online tool (www.randomizer.org). One tooth for each group received only the enamel conditioning treatment and, under scanning electron microscope (SEM) imaging, was used to characterize the morphology of the treated surface, leaving ten teeth for each group for the measurements of shear debonding force.

### 2.1. Enamel Conditioning

The three groups of teeth received a different procedure for enamel conditioning prior to the bonding of a bracket on the buccal surface of the crown:

The Etching group (E group, *n* = 10) received a conditioning with the application of 37% phosphoric acid gel (DentoEtch, Itena, Paris, France) for 30 s, then was rinsed with distilled water for 30 s and accurately dried with compressed air. The gel was applied on an area of approximately 5 × 3 mm. This group acted as a control for the other two where procedures different from the standard were performed.

The Hydroabrasion group (HA group, *n* = 10) received a conditioning with a hydroabrasion device (PrepStart H_2_O, Danville, CA, USA) at an air pressure of 3 bar (43.5 psi), the flow of 27 μm aluminum oxide powder set at minimum flow, and the water flow opened by two turns and four graduation marks; the nozzle of the handpiece was oriented perpendicularly to the enamel surface, at a distance of nearly 5 mm, for 5 s on an area of approximately 5 × 3 mm.

The Hydroabrasion and Etching group (HA-E group, *n* = 10) first received a conditioning with hydroabrasion, with the same methodology used for the HA group, followed by an acid etching with the same methodology described for the E group.

One tooth from each of the three groups was observed with a field emission gun SEM (GeminiSEM 500, Zeiss Microscopy, Jena, Germany) to describe the morphologic characteristic of the enamel following each type of conditioning. After application of a thin (5 nm) conductive coating (chromium, Cr) to the tooth surface (Q150T ES, Quorum Technologies Ltd., Laughton, UK), the specimen was observed at 100×, 500×, 5000×, and 10,000× magnification, using the signal from secondary electrons (SEs) to obtain a clearer view of the surface topography.

### 2.2. Bonding and Sample Preparation

**Figure 1:**
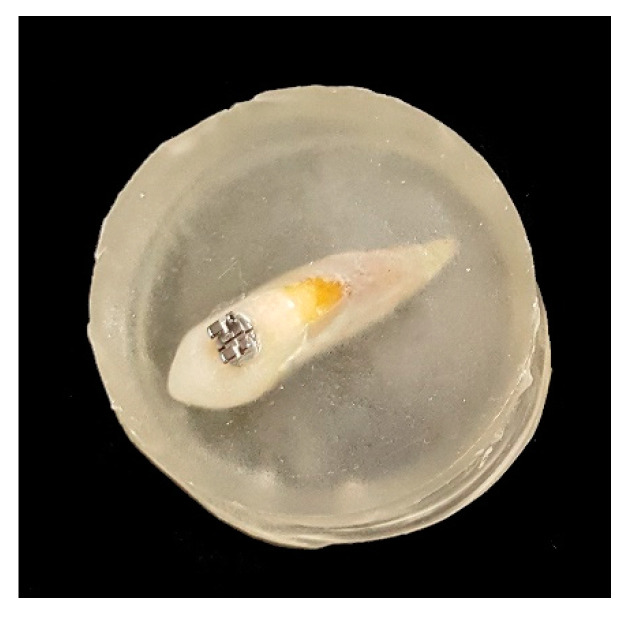
A tooth with a metal bracket bonded on, embedded in acrylic resin for the mechanical testing of debonding force.

The prepared teeth were embedded in a self-curing acrylic resin (Orthojet, Lang Dental Mfg Co. Inc., Wheeling, IL, USA) using a 30 mm diameter round silicon mold, taking care to have the dental surface with the bracket bonded parallel to the ground and not covered by the resin (Figure 1). After an initial setting time of 2 min, the acrylic resin was cured for an additional 10 min in water at 30 psi, according to the manufacturer’s instruction. The samples were then extracted from the mold, refined and polished.

An upper premolar orthodontic bracket (Midi diagonali, Leone S.p.a., Sesto Fiorentino, Italy) was bonded on the buccal surface of the 30 teeth. A thin layer of adhesive primer (Transbond XT Light Cure Adhesive Primer, 3M Unitek, Monrovia, CA, USA) was coated above the conditioned surface; the excess was gently removed with compressed air and suction, then light-cured for 10 s at 1200 mW/cm^2^ (Bluephase^®^ G4, Ivoclar Vivadent, Schaan, Liechtenstein). A layer of composite (Transbond XT, 3M Unitek, Monrovia, CA, USA) was applied to the meshed base of the bracket, which was subsequently firmly seated on the buccal surface of the tooth, at the center of the facial axis of the crown. After removing the excess with a dental explorer, the composite was light-cured for 20 s at 1200 mW/cm^2^ (Bluephase^®^ G4, Ivoclar Vivadent, Schaan, Liechtenstein).

### 2.3. Shear Debonding Force Testing

**Figure 2:**
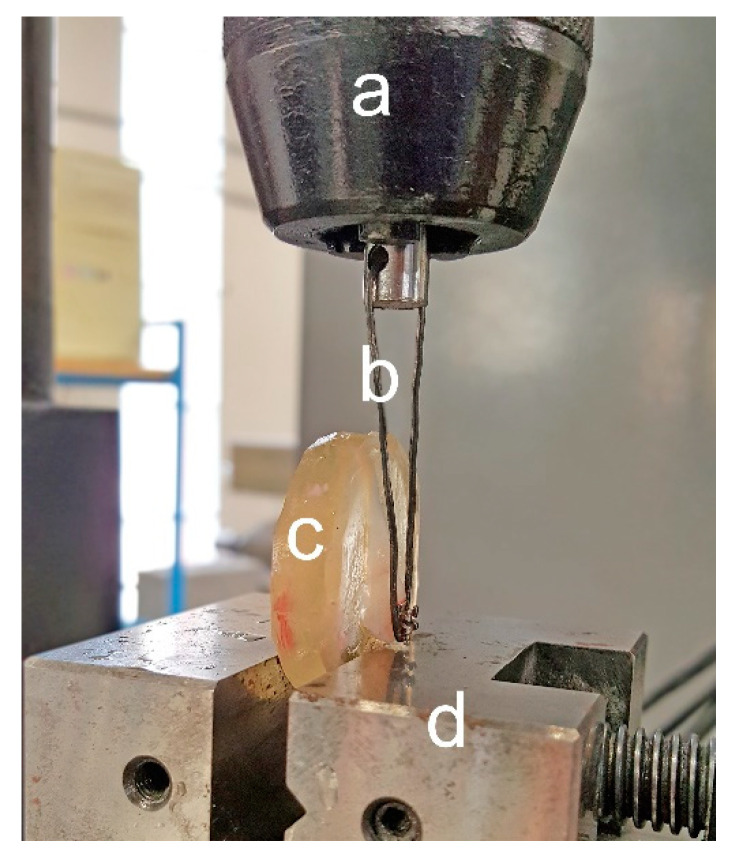
Schematic representation of the experimental setting for testing of shear bond strength. (**a**) Load cell; (**b**) metal loop that connects the bracket to the load cell; (**c**) embedded specimen secured to a vice (**d**).

The experimental apparatus consisted of a precision traction testing machine with automatic axial feeding, controlled by a personal computer via a D/A card. The resin blocks were secured into a vice, the tooth and bracket surface being perfectly perpendicular to the ground, and at the exact center of the load cell. The load cell and the bracket were connected through a rigid metal loop, which embraced the occlusal surface of the bracket, without touching its wings (Figure 2). The load cell was set to run at a crosshead speed of 1 mm/min until failure of the bracket-to-enamel bonding. Force values were detected by means of a piezoelectric load cell (9065 SN 3786, Kistler Group, Winterthur, Switzerland) with a sensor sensitivity of −2.02 pC/N. The signal was conditioned by charge amplifiers, filtered by an anti-aliasing analogue filter and acquired by the computer via the A/D card. The load curve was measured for every sample, and the maximum force level (N) recorded before breakage was stored for further analysis. Knowing the surface of the bracket’s base (11 mm^2^), this value was also reported as pressure (N/mm^2^). The number of enamel fractures after debonding was also recorded.

### 2.4. Adhesive Remnant Index

After force testing, the enamel surface of each of the samples was observed under a stereomicroscope (S8 APO, Leica Microsystems, Wetzlar, Germany) equipped with a digital colour camera (EC3, Leica Microsystems, Wetzlaar, Germany). The presence of adhesive/composite residuals was evaluated on 60× images with the adhesive remnant index (ARI) as proposed by Årtun and Bergland [19], assigning scores from 0 to 3 to each tooth according to the following criteria: score 0 = no adhesive left on the tooth; score 1 = less than half of the adhesive left on the tooth; score 2 = more than half of the adhesive left on the tooth; score 3 = all adhesive left on the tooth, with distinct impression of the bracket mesh. The scores were calculated twice at a two-week interval by one operator (MT) who was blinded regarding the method used for enamel conditioning on each surface. Cohen’s weighted kappa was calculated on the two sets of scoring to evaluate the intra-observer agreement.

### 2.5. Statistical Analysis

Descriptive statistics were calculated for all the variables. The force and pressure measurements were tested for a normal distribution with a Shapiro−Wilk normality test. A one-way ANOVA was used to evaluate the presence of difference in force and pressure values between the three groups. A Tukey’s or Games−Howell post hoc test was subsequently calculated, depending on data homoscedasticity, as evaluated with a Levene’s test.

A Kruskal−Wallis H test was computed to evaluate the presence of difference in ARI scores between the three groups. Post hoc pairwise comparisons were performed using a Mann−Whitney U test. First-type error was set at *p* = 0.05 for all the tests.

## 3. Results

**Figure 3:**
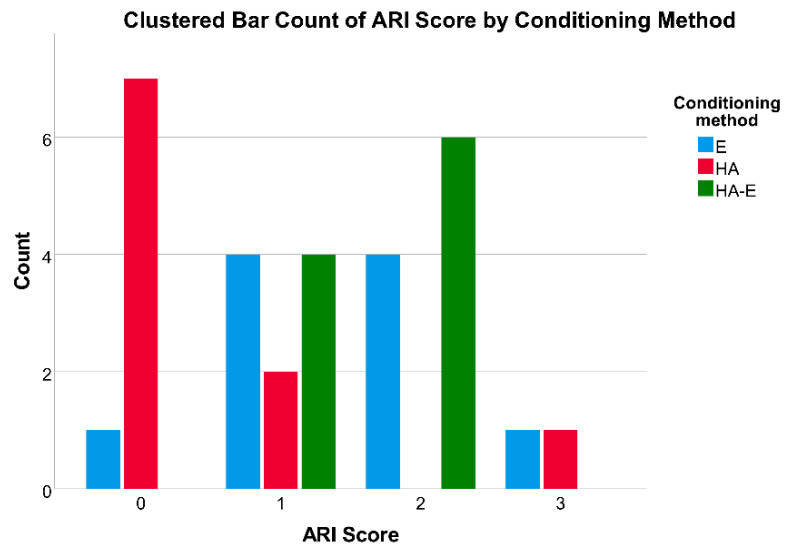
Histogram reporting the frequency of ARI scores for the acid etching (E) group, for the hydroabrasion group (HA), and for the hydroabrasion and acid etching (HA-E) group. ARI, adhesive remnant index according to Årtun and Bergland; Score = 0 no adhesive left on the tooth; Score 1 = less than half of the adhesive left on the tooth; Score 2 = more than half of the adhesive left on the tooth; Score 3 = all adhesive left on the tooth, with distinct impression of the bracket mesh.

Descriptive statistics for force and pressure values are reported in Table 1, while ARI score frequencies are reported in Figure 3. Intra-rater agreement as calculated with Cohen’s kappa with square weights was 99.4% for ARI scores (kappa = 0.95; *p* < 0.001).

**Figure 4:**
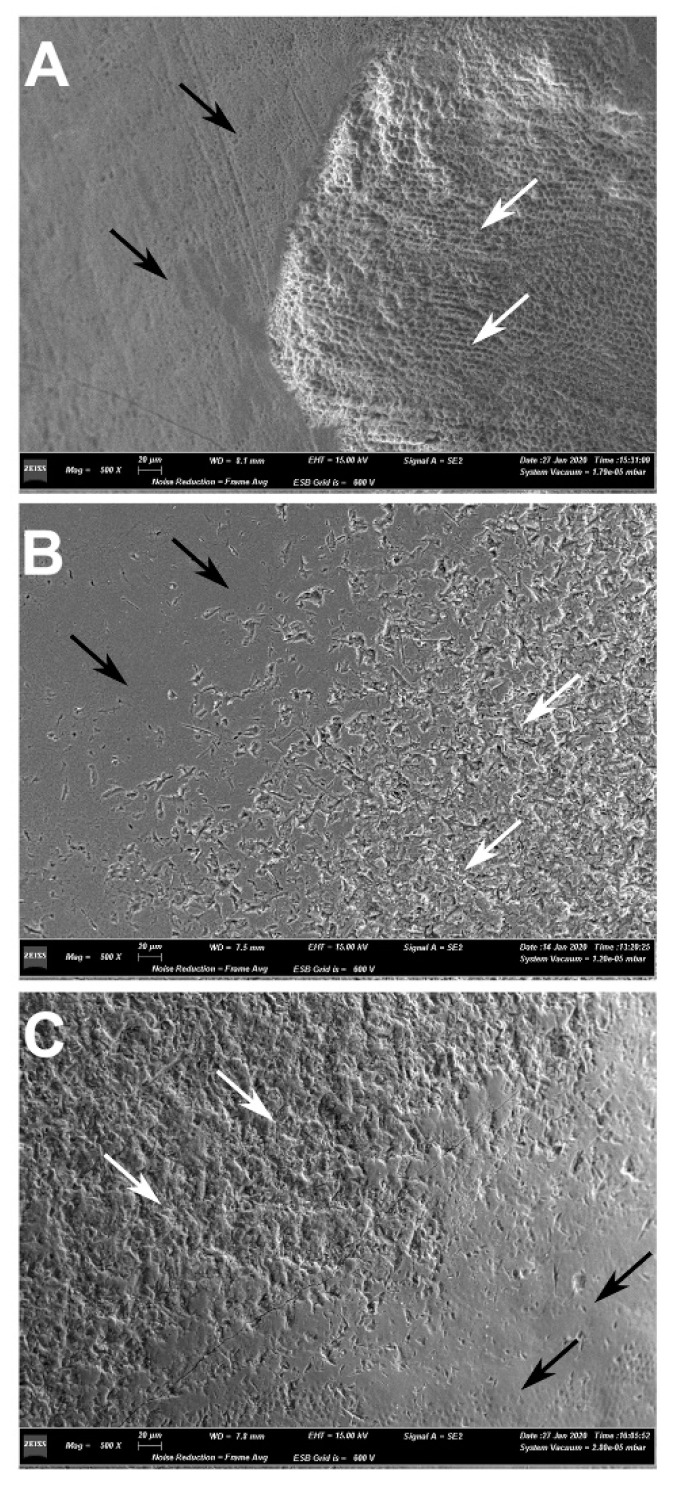
Scanning electron microscope (SEM) imaging of the teeth after application of different enamel conditioning treatments. Images were taken at 500× magnification under SE modality at high tension (15.0 kV), depicting the interface between the intact enamel and the conditioned surface. (**A**) acid etching (E group); (**B**) hydroabrasion (HA group); (**C**) acid etching and hydroabrasion (HA-E group); black arrows, intact enamel; white arrows, conditioned enamel.

**Figure 5:**
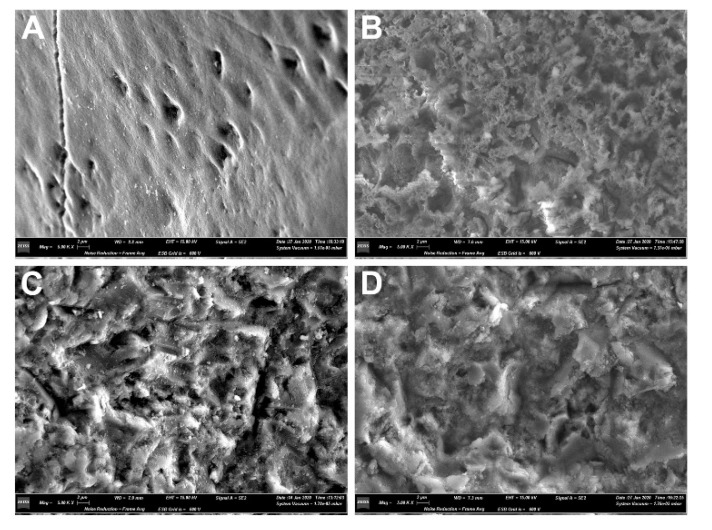
Scanning electron microscope (SEM) imaging of the teeth after application of different enamel conditioning treatments. Images were taken at 5000× magnification under SE modality at high tension (15.0 kV). (**A**) normal and intact enamel; (**B**) acid etching (E group); (**C**) hydroabrasion (HA group); (**D**) acid etching and hydroabrasion (HA-E group).

The morphology of the enamel surface after conditioning with the three methods can be observed in Figure 4 and Figure 5.

**Figure 6:**
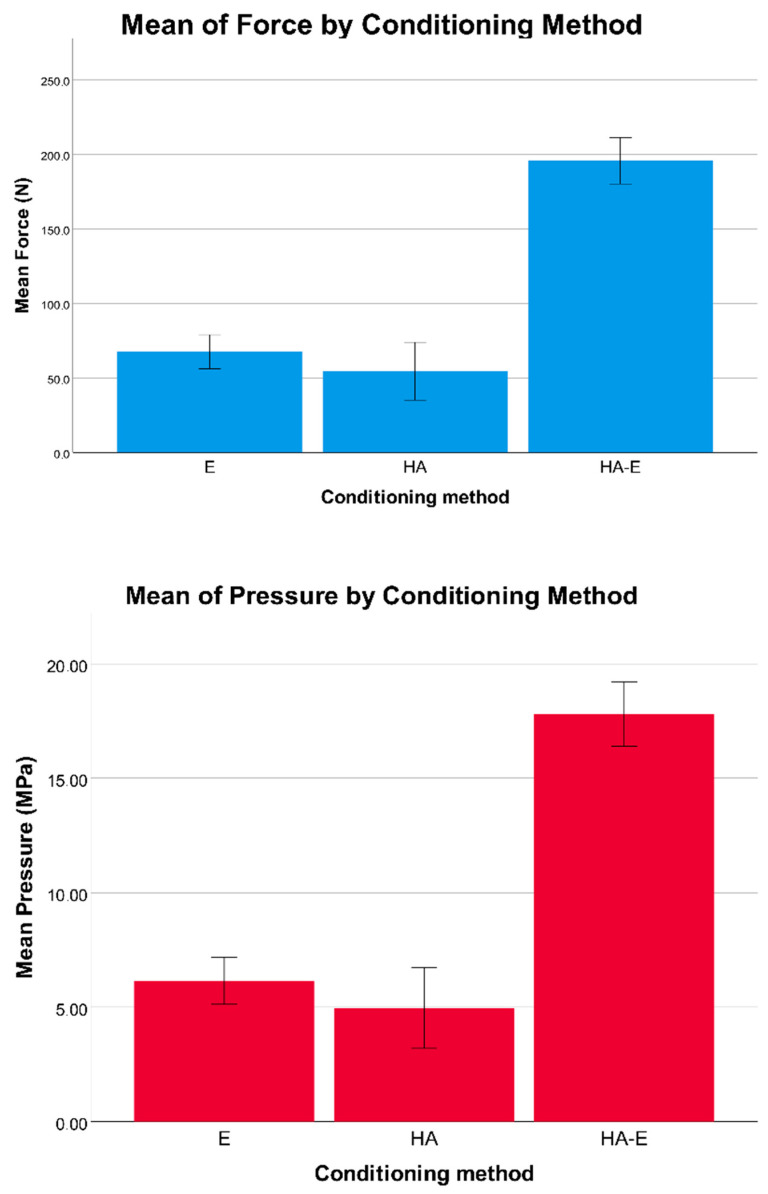
Histograms reporting the mean values of shear bond force (N) and pressure (MPa) respectively, for the acid etching (E) group, for the hydroabrasion group (HA), and for the hydroabrasion and acid etching (HA-E) group. The 95% confidence interval is reported as error bars.

One-way ANOVA revealed a statistically significant difference for force and pressure debonding values between the three groups (Table 2, Figure 6). In detail, the post hoc test revealed that enamel conditioning in the HA-E group produced significantly higher debonding force and pressure values than the E group and the HA group (Table 3). In addition, one tooth in the E group and one in the HA group presented enamel fractures when observed after the shear bond testing, while five teeth in the HA-E group presented enamel fractures, even if these data were not tested statistically.

**Figure 7:**
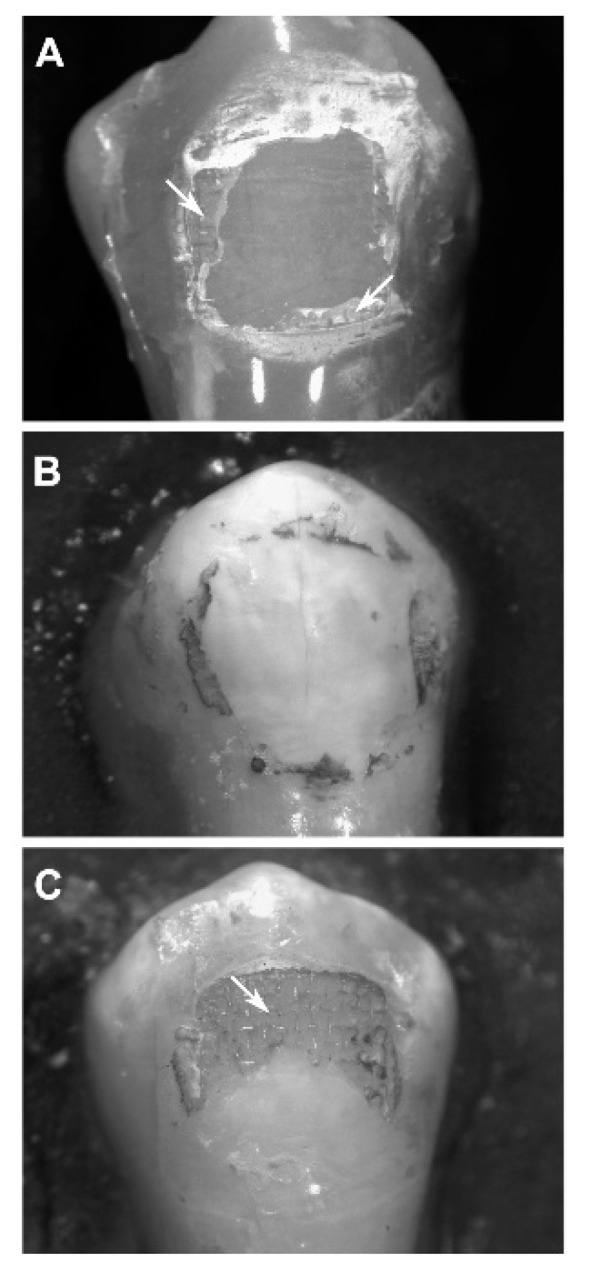
Stereomicroscope imaging at 60× magnification of the tooth samples. White arrows, composite remnants. (**A**) sample from the E group, showing an adhesive remnant index (ARI) score of 1; (**B**) sample from the HA group, showing an ARI score of 0; (**C**) sample from the HA-E group, showing an ARI score of 2.

Regarding the ARI scores (Figure 7), a Kruskal−Wallis H test showed that there was a statistically significant difference between the different enamel conditioning treatments, χ^2^(2) = 9.692, *p* = 0.008, with a mean rank ARI score of 18.2 for the Etching group, 8.8 for the Hydroabrasion group and 19.5 for the Etching and Hydroabrasion group. The ARI scores were significantly different between the E group and the HA group, and between the HA group and the HA-E group (Table 4). Therefore, the null hypothesis was rejected.

## 4. Discussion

Enamel conditioning is a necessary procedure for composite bonding. The gold standard technique is acid etching, whose primary effect is to increase the contact area by roughening the enamel surface, and to modify a low-energy hydrophobic surface into a high-energy hydrophilic surface [20]. A low-viscosity resin (i.e., the adhesive) is then applied to the surface, infiltrating the micropores left in the prismatic and interprismatic enamel by the action of the etchant: the micromechanical interlocking between the roughened enamel and the low-viscosity resin is the mechanism that underlies enamel bonding [21]. The composite can then bond chemically to the adhesive layer. This procedure, however, necessarily leads to a certain amount of enamel substance loss, ranging from 10 to 80 μm, and represents a form of iatrogenic damage [3]. Observing the effects on the enamel surface, it is possible to recognize from an overview (Figure 4A) the typical honeycomb pattern produced by acid etching, which reveals the presence of small and irregular grooves at a greater magnification (Figure 5B). Hydroabrasion with 27 μm particles produced indentations with a pyramidal shape (Figure 4B and Figure 5C), which, although they look deeper at some points, have a wide base and leave larger areas with a regular surface. The combination of the two procedures resulted in a more complex pattern, with deep and wide depressions roughened by the action of the phosphoric acid (Figure 4C and Figure 5D). Some authors [3] observed a comparable enamel loss between etching and air abrasion at low pressure (1 bar) and short application (1 s), but in our study, the longer time and higher pressure produced a greater substance loss. In another study, the authors using air abrasion with larger particles (50 μm) at 4.8 bar for 3 s observed no statistically significant differences in surface roughness compared to traditional methods [14]. Some authors reported that etching with phosphoric acid removes only the inorganic part of the enamel, while the organic matrix is left intact—and this is a difference compared to air abrasion or hydroabrasion—and can be remineralized, thus resulting in a minimal net loss of enamel [22]. However, this is true only if the etched enamel receives no further treatment: indeed, if a layer of resin adhesive is applied to it, then the choice is between accepting leaving adhesive remnants on the tooth surface or removing that amount of enamel together with the adhesive, as demonstrated by different studies [18,21].

These morphologic characteristics are reflected in the shear bond strength values. When a bracket is removed by the operator or due to failure, debonding can occur at different levels: at the bracket/composite interface (adhesive failure), at the adhesive/enamel interface (adhesive failure), within the adhesive layer (cohesive failure), or at different levels at the same time (mixed failure) [23]. Different types of bonding failure result in different amounts of adhesive/composite on the enamel surface. To correctly interpret these findings, it is first necessary to recall the clinical requirements outlined by Reynolds [19]: considering that bite forces are on average between 40 and 120 N, a minimum of 6 MPa is recommended for orthodontic bracket bonding. On the other hand, the cohesion of enamel itself is between 9 and 11 MPa, so this should be considered as the upper limit, to prevent an increased risk of enamel fractures [24].

According to the results of the present study, mechanical roughening through hydroabrasion alone is not sufficient to provide clinically meaningful bracket adhesion, since the shear bond strength values measured were below 6 MPa. This is probably explained by the enamel morphology that is obtained, where deep but wide grooves are not able to provide enough mechanical interlocking between tooth and adhesive resin. The results of the shear bond test were confirmed by the ARI scores, which revealed how almost all the debondings were at the interface between resin and enamel (Table 4, Figure 3 and Figure 7). This is in accordance with the results of previous studies [10,12,22], even though it is not easy to compare the present data with the existing literature, because hydroabrasion is a different technology to air abrasion and was not tested before, and because a variety of pressure levels, particle sizes, operating distances, angulations and application times were used.

On the other hand, the combination of etching and hydroabrasion led to significantly higher shear bond strength values of around 17 MPa in the HA-E group. This value is beyond the limit of the enamel’s internal cohesion; however, a bracket in vivo is never debonded with a pure shear force, which would increase the possibility of tooth fracture, but is usually achieved through a peeling movement resulting from a combination of shear, tensile and torsional forces [14,25]. However, some enamel fractures were observed in the HA-E group. Brauchli et al. [12] reported similar values of 16.5 MPa with the same resin composite used in the present study (Transbond^TM^ XT) after enamel conditioning with air abrasion with 50 μm aluminum oxide and acid etching; Halpern et al. [11] measured 8.7 MPa with 50 μm particles and 10.2 MPa using 100 μm particles at 4.8 bar for 1 s; Canay et al. [10] drew the same conclusion that etching and air abrasion together produce the highest force, although reporting smaller absolute values (6.1 MPa using 50 μm aluminum particles at 5.5 bar, applied for 5 s at 10 mm distance with 45° angulation). In contrast, a few studies [16,26,27] reported no effect of using 50 μm particles in air abrasion on orthodontic bonding. Interestingly, Nikaido et al. [28] reported a decreased bond strength when using air abrasion with 50 μm aluminum oxide, compared to etching alone. Some authors that used air abrasion with 25 μm aluminum oxide reported no significant difference compared to etching alone [27,29,30], in contrast to Mujdeci and Gokay [31], who reported an increased bond strength, underlining again the importance of the pressure/particle size/time/distance settings.

One last consideration should be made regarding the safety of hydroabrasion. There are available data only regarding air abrasion: if aimed directly towards the gingiva, epithelial erosive changes can be observed [32], even though, according to the law of kinetic energy, cutting is faster on harder tissues and slower on softer objects due to the energy that is lost because of the tissue’s resilience, therefore the gingiva and the mucosa are fairly protected [33,34]. Wright et al. [35] evaluated the health hazard to patients and operators of air abrasion, concluding that their findings were insufficient to demonstrate the presence of any health-related risk. Moreover, hydroabrasion could be considered safer since the powder flow is contained by the water spray [17], even though further studies are needed to prove this aspect.

Regarding the limitations of the present study, in vitro studies are unable to perfectly replicate the conditions that are present in an in vivo environment, therefore these kinds of results should be interpreted with caution. In addition, shear bond strength is only one of the components that contribute to the clinical efficacy of the orthodontic bracket bonding.

## 5. Conclusions

(1) All the tested enamel conditioning methods produce a certain amount of permanent enamel loss.
(2) Hydroabrasion alone is not an adequate method to achieve a clinically meaningful bonding strength.
(3) Hydroabrasion followed by acid etching results in higher shear bond strength, but seems to be followed by an increased risk of enamel fracture. Further in vivo studies are needed to carefully evaluate the cost−benefit ratio of this technique for improving bracket bond strength.

## Figures and Tables

**Table 1:** Descriptive statistics for debonding force and pressure (*n* = 10).

	Etching	Hydroabrasion	Hydroabrasion and Etching
Force (N)	67.1 ± 10.5	55.5 ± 20.6	195.7 ± 19.1
Pressure (N/mm^2^)	6.1 ± 0.9	5.0 ± 1.9	17.8 ± 1.7

Mean ± Standard deviation.

**Table 2:** One-way ANOVA for force and pressure values between groups (*n* = 10).

Variable	Homogeneity of Variances	Sum of Squares	Mean Square	F	*p*
Force	0.319 ^†^	121,184.8	60,592.4	201.7 **	<0.001
Pressure	0.321 ^†^	1001.4	500.7	201.9 **	<0.001

** statistically significant with *p* < 0.01; ^†^ *p* value from Levene statistic.

**Table 3:** Tukey’s post hoc comparison between groups (*n* = 10).

Dependent Variable	Group (I)	Group (J)	Mean Difference (I-J)	SE	*p*	95% Confidence Interval	
						Lower Bound	Upper Bound
Force (N)	Etching	Hydroabrasion	11.6	7.7	0.308	−7.6	30.8
		Hydroabrasion and Etching	−128.6 **	7.7	<0.001	−147.9	−109.4
	Hydroabrasion	Hydroabrasion and Etching	−140.2 **	7.7	<0.001	−159.5	−121.0
Pressure (N/mm^2^)	Etching	Hydroabrasion	1.1	0.7	0.307	−0.7	2.8
		Hydroabrasion and Etching	−11.7 **	0.7	<0.001	−13.4	−9.9
	Hydroabrasion	Hydroabrasion and Etching	−12.7 **	0.7	<0.001	−14.5	−11.0

** statistically significant for *p* < 0.01.

**Table 4:** Post hoc pairwise comparisons of adhesive remnant index (ARI) scores frequency between the groups (*n* = 10).

	Mann-Whitney U	*p* Value
Etching vs. Hydroabrasion	19.0 *	0.014
Etching vs. Hydroabrasion and etching	46.0	0.737
Hydroabrasion vs. Hydroabrasion and etching	14.0 **	0.004

* statistically significant for *p* < 0.05; ** statistically significant for *p* < 0.01.
